# Desmoplastic Fibromas of the Bone: A Systematic Review of Clinical Presentation and Surgical Treatment

**DOI:** 10.3390/cancers17213558

**Published:** 2025-11-03

**Authors:** Edoardo Ipponi, Francesco Pecchia, Sahar Toumie, Antonio D′Arienzo, Paolo Domenico Parchi, Lorenzo Andreani

**Affiliations:** Department of Orthopedics and Trauma Surgery, University of Pisa, Via Paradisa 2, 56124 Pisa, Italy

**Keywords:** tumor, curettage, resection, local recurrence, symptoms, pathological fracture

## Abstract

**Simple Summary:**

Desmoplastic fibroma is a rare, benign, but locally aggressive bone tumor. First described in 1958, to date, only a limited number of cases are available, and little is known about the clinical presentation and outcomes of surgical treatment. Our review included 187 cases of desmoplastic fibroma treated with surgery. The most frequently involved bones were the femur, the mandible, and the pelvis. Pain and swelling were common findings. Pathological fractures occurred in 11% of cases. Bone resections (112) and curettage (70) were the most common surgical treatments. Curettage was associated with significantly higher local recurrence rates (38.5) compared to bone resections (11.6) during the post-operative follow-up.

**Abstract:**

Background: Bone desmoplastic fibroma (DF) is a rare, locally aggressive, benign tumor. Due to its low incidence, studies on the topic have been limited to case reports and a few case series. This review aims to summarize modern literature on bone DF and provide an overview of its clinical presentation and prognostic horizons after surgical treatment. Methods: We systematically searched for articles reporting on DFs treated surgically. Our research was conducted according to the PRISMA guidelines, including PubMed, Embase, and Scopus articles between 1958 and 2025. Lesions’ location and size, tumors’ symptoms, the surgical treatment of choice, and the recurrence rates at patients’ latest follow-up were recorded. Results: A total of 97 articles and 187 cases were included. The mean age was 24.3. There was no gender difference. The lower limb was the most common localization (87 cases; 47%), followed by the upper limb (48; 26%), mandible (27; 14%), spine (17; 9%), and other sites (8; 4%). Pain was detected in 73% of cases and swelling in 53%. Seventeen patients (11%) had pathological fractures. Focal resections and curettage were the most common surgical treatments. Thirty-eight cases (23%), most treated with intralesional curettage, had local recurrence after a mean follow-up of 63 months. Conclusions: Despite its low incidence, desmoplastic fibromas should be considered in the differential diagnosis of symptomatic osteolytic bone lesions. Although curettage can be considered as a reasonable solution for selected cases, wide resections are recommended when feasible to minimize the risk of local recurrence.

## 1. Introduction

Desmoplastic fibromas of the bone are rare, benign but locally aggressive tumors characterized by intraosseous proliferation of spindle-shaped fibroblasts embedded in a dense collagenous matrix [1]. The tumor was first formally described in 1958 by Jaffe, who recognized its similarity to extra-abdominal desmoid tumors and proposed that it represented the osseous counterpart of soft tissue fibromatosis [2]. Since that time, more cases have been reported in the literature, progressively increasing and gradually laying the foundation for current knowledge on this topic. Desmoplastic fibroma is now included in the latest WHO classification of bone and joint tumors as a fibrogenic neoplasm with intermediate behavior [3]. While some parallels with desmoid-type fibromatosis remain, desmoplastic fibroma has come to be recognized as a distinct entity due to singular oncological behavior and peculiar clinical implications [1,2,3,4,5].

Epidemiologically, desmoplastic fibromas are rare tumors, accounting for only 0.1 to 0.3 percent of all primary bone tumors and less than 1 percent of benign bone lesions. The total number of cases reported worldwide remains in the low hundreds, making it one of the rarest benign bone tumors [5,6,7,8]. Due to their rarity, desmoplastic fibromas can be challenging to consider in the differential diagnosis and may be difficult to identify when encountered in neoplastic bone lesions. As with most bone tumors, the diagnostic pathway relies on a combination of clinical, radiographic, and histopathological assessments [9]. 

**Figure 1:**
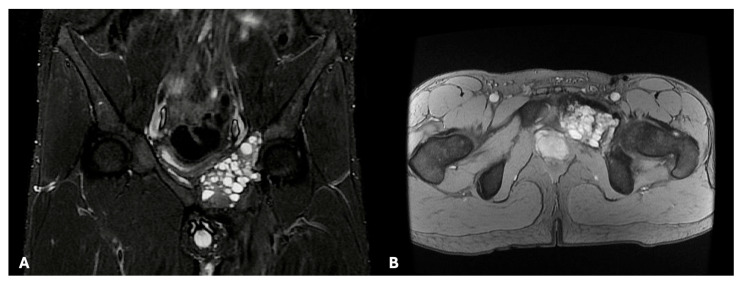
Coronal ((**A**); STIR sequence) and axial ((**B**); MRGE sequence) views of a desmoplastic fibroma of the right pubis and iliopubic branch. In both images, the lesion presents a multi-cystic appearance.

In the absence of any known pathognomonic signs or symptoms, bone tumors such as desmoplastic fibromas can present with localized pain, swelling, or restricted function, depending on the lesion’s site and size. Occasionally, pathological fractures may be the first manifestations of the disease. In some other instances, tumors can stay clinically silent and be detected accidentally while performing radiological exams for contingent reasons. Still, little is known about the clinical characteristics and pathological fracture rates of desmoplastic fibromas in particular, due to their extreme rarity [5,8]. Radiographs usually reveal desmoplastic fibromas as lytic masses with an invasive growth pattern rather than an expansive one. Radiographically, a desmoplastic fibroma appears as a well-defined, geographic, lytic (bone-destroying) lesion with a narrow zone of transition, being generally classified as grade 1 lesions according to the Lodwick and Madewell classification. In some cases, lesions may also expand the host bones, leading to cortical thinning or scalloping [9,10,11]. Computed tomography can also be valuable for assessing cortical disruption and eventual extraosseous extension, whereas magnetic resonance provides superior detail on the fibrous appearance of the tumor (Figure 1).

Imaging evidence can orient the differential diagnosis between desmoplastic fibromas and other fibrous benign bone lesions, such as non-ossifying fibromas, fibrous dysplasia, aneurysmal bone cysts, and chondromyxoid fibroma, as well as other locally aggressive bone tumors such as giant cell tumors of the bone.

Although imaging evidence can orient the presumptive diagnosis towards solid locally aggressive bone lesions, a biopsy is necessary to establish a diagnosis of desmoplastic fibroma of the bone [10,11,12,13,14,15,16,17]. Histologically, the tumor is composed of bland spindle fibroblasts in a dense collagenous background. Mitotic figures are scarce, and cytological atypia is low or absent [18,19,20,21,22,23]. Desmoplastic fibroma of bone is driven by slow but infiltrative growth, without proven metastatic potential. Immunohistochemical studies have shown cytoplasmic and occasional nuclear β-catenin expression. Immunohistochemical β-catenin expression may overlap with desmoid-type fibromatosis but typically lacks consistent nuclear localization. Some cases exhibit somatic CTNNB1 mutations similar to those observed in desmoid tumors, suggesting a shared pathogenesis. Other reported genetic findings include rearrangements involving 11q13, although recurrent, disease-defining alterations remain unproven [21,24,25,26,27].

Once the diagnosis has been established, appropriate treatment should be initiated to achieve local control and disease eradication. Although pharmacological and radiant treatments have been described as stand-alone options for selected cases of desmoplastic fibroma of the bone, surgery remains the treatment of choice for most cases [28,29]. Different surgical approaches have been described and performed, depending on the size and location of the individual lesions, primarily following the common principles of treatment for benign but locally aggressive bone tumors [1,3,17,30].

To date, the actual effectiveness of these various surgical treatments in terms of tumor eradication and local disease control remains largely unestablished. The low incidence of desmoplastic fibromas of the bone, and consequently, the limited number of case reports and even fewer case series described in the literature, provide only limited data on the topic. 

Our review aims to summarize the modern literature on desmoplastic fibromas and increase orthopedic oncologists’ knowledge of this topic. From a pre-operative perspective, particular focus was placed on patients’ clinical presentation, as a better understanding of the disease’s signs and symptoms could facilitate and enhance the diagnostic pathway. The oncological outcomes of surgical treatments and the risk of local recurrence were also evaluated to assess the reliability of these treatments.

## 2. Materials and Methods

A systematic review of the literature was performed according to the Preferred Reporting Items for Systematic Reviews and Meta-Analyses (PRISMA) guidelines, using the Supplementary Material and algorithm. The systematic review has not been registered (Appendix A). A comprehensive search of the PubMed, MEDLINE, EMBASE, and Scopus databases using various combinations of the keywords “Desmoplastic fibroma,” “bone”, and “surgery,”. We included papers published between 1958 and 2025, available as of 1 September 2025.

All the original articles reporting on patients diagnosed with desmoplastic fibromas of the bone that required surgical treatment were included. Three independent reviewers (E.I., F.P., S.T.) conducted the research separately. Only articles from peer-reviewed journals were included. The investigators separately reviewed each publication’s abstract and then closely read all articles, extracting data to minimize selection bias and errors.

Inclusion criteria were (1) a confirmed histological diagnosis of desmoplastic fibroma partially or completely involving a bone, (2) a surgical treatment aimed to eradicate or control the disease, and (3) follow-up information regarding the oncological and, eventually, clinical outcomes after surgical treatment. Exclusion criteria were (1) articles that did not mention or provide data on the surgical treatment of the postoperative outcome, (2) a follow-up shorter than six months, (3) pre-clinical studies, (4) literature reviews without any new cases, and (5) papers written in languages other than English. 

**Figure 2:**
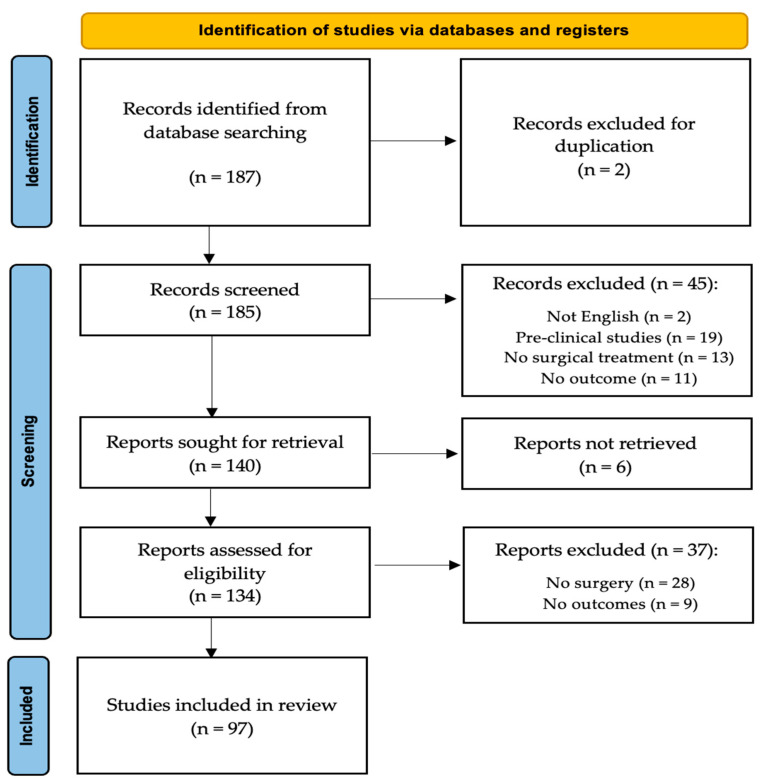
A schematic representation of our study’s PRISMA flow-chart.

All articles were initially screened for relevance by title and abstract, excluding articles without an abstract, and obtaining the full-text article if the abstract did not allow the investigators to assess the presence of inclusion and exclusion criteria. Considering the limited number of large-sized case series articles and the low level of evidence in the few available, we included in our study articles ranging from Level I to Level V, as well as detailed case reports. The search algorithm, as outlined in the PRISMA guidelines, is presented in Figure 2. The review was not registered.

For each article, we reported the year of publication and the article type, distinguishing between case reports and case series. The number, age, and gender of patients were recorded for each article. Symptoms, when reported, were also documented, with a particular focus on pain and local swelling. The involved bone and the lesion’s size were also noted, as well as eventual pathological fractures. For each case, we recorded the surgical treatment of choice and whether they had a post-operative local recurrence or not. The follow-up of each case report and the mean follow-up of all the case series were noted. Percentages and rates of the individual items were calculated based on the articles reporting on those items.

**Figure 3:**
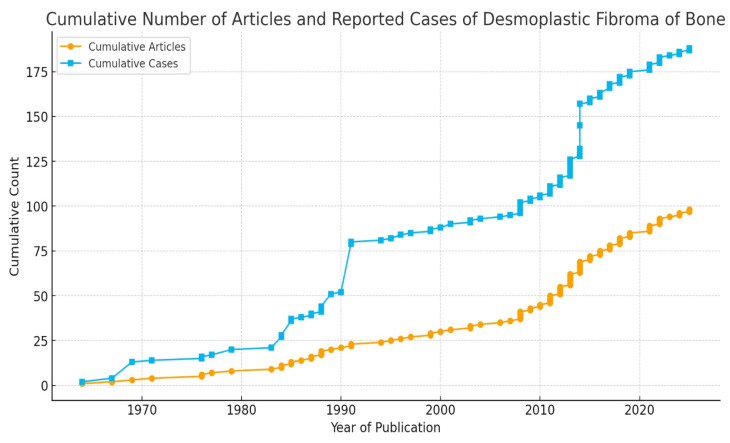
Graphic representation of the cumulative number of published articles (orange line) and reported cases (blue line) until 2025.

Ninety-seven articles met our inclusion and exclusion criteria and were included in our review [1,3,5,7,11,12,13,14,15,16,17,18,20,21,22,23,30,31,32,33,34,35,36,37,38,39,40,41,42,43,44,45,46,47,48,49,50,51,52,53,54,55,56,57,58,59,60,61,62,63,64,65,66,67,68,69,70,71,72,73,74,75,76,77,78,79,80,81,82,83,84,85,86,87,88,89,90,91,92,93,94,95,96,97,98,99,100,101,102,103,104,105,106,107,108]. There were eighteen case series [1,3,5,13,17,30,31,32,33,34,35,36,37,38,39,40,41] and seventy-nine case reports [7,11,12,14,15,16,18,20,21,22,23,42,43,44,45,46,47,48,49,50,51,52,53,54,55,56,57,58,59,60,61,62,63,64,65,66,67,68,69,70,71,72,73,74,75,76,77,78,79,80,81,82,83,84,85,86,87,88,89,90,91,92,93,94,95,96,97,98,99,100,101,102,103,104,105,106,107,108]. The yearly distribution of articles is portrayed in Figure 3. As no randomized trial was included, bias assessment for randomized studies was not performed.

To account for heterogeneity in study design and methodology across the selected cohort studies and case series, the Joanna Briggs Institute (JBI) Critical Appraisal tools were used to assess their quality for inclusion in this systematic review. Each item on the checklist is rated with one of four possible responses: “yes”, “no”, “unclear”, or “not applicable” [109].

## 3. Results

Ninety-seven articles met our inclusion criteria. Among them, seventy-nine were case reports and eighteen were case series. A total of 187 cases were included in our review [1,3,5,7,11,12,13,14,15,16,17,18,20,21,22,23,30,31,32,33,34,35,36,37,38,39,40,41,42,43,44,45,46,47,48,49,50,51,52,53,54,55,56,57,58,59,60,61,62,63,64,65,66,67,68,69,70,71,72,73,74,75,76,77,78,79,80,81,82,83,84,85,86,87,88,89,90,91,92,93,94,95,96,97,98,99,100,101,102,103,104,105,106,107,108]. A report of all the case series and a summary of all the case reports included in our review are reported in Table 1.

### 3.1. Quality Assessment

Due to size limitations, none of the included studies provided statistical analyses. Therefore, Q10 was designed as not available in all of them.

Nine of the 18 case series articles included in our review had yes to all the remaining queries of the JBI checklist. The remaining nine articles were found to be unclear in at least one of the areas investigated by the checklist, but were still considered worthy of being maintained in our review. The JBI quality assessment of all the included articles was reported in detail in Table 2.

### 3.2. Demographics

**Figure 4:**
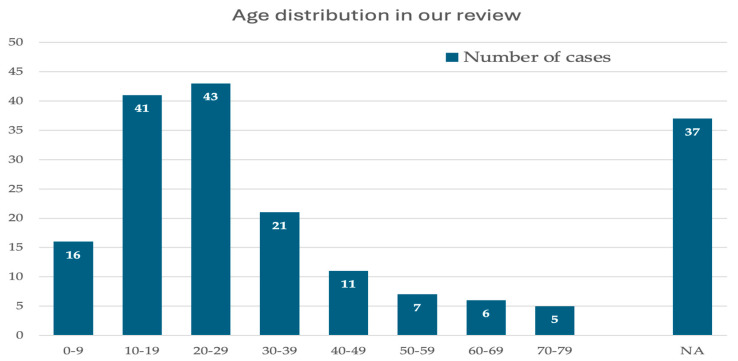
A graphic distribution of patients, sorted per their age groups (decades). On the right, those cases whose age was not specified (NA).

A total of 187 cases, 104 males (55.8%) and 83 females (44.2%), were included in our analysis. A binomial test did not reveal a statistically significant gender discrepancy among the evaluated patients (*p* = 0.5266). Patients’ mean age was 24.3 (2–79). The second and third decades of life were the most common at diagnosis (Figure 4) [1,3,5,7,11,12,13,14,15,16,17,18,20,21,22,23,30,31,32,33,34,35,36,37,38,39,40,41,42,43,44,45,46,47,48,49,50,51,52,53,54,55,56,57,58,59,60,61,62,63,64,65,66,67,68,69,70,71,72,73,74,75,76,77,78,79,80,81,82,83,84,85,86,87,88,89,90,91,92,93,94,95,96,97,98,99,100,101,102,103,104,105,106,107,108].

### 3.3. Anatomical Distribution

**Figure 5:**
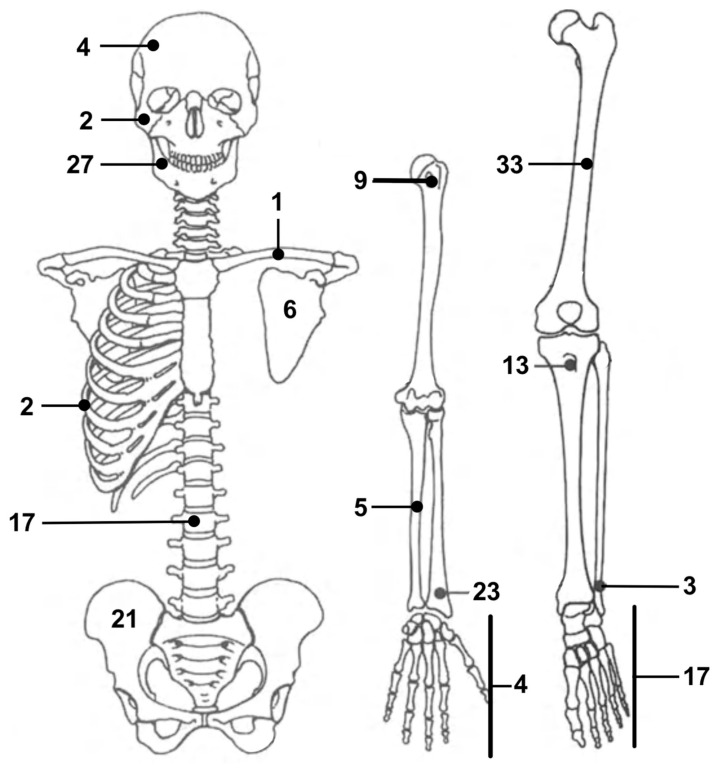
Schematic distribution of desmoplastic fibromas of the bone included in our literature review.

None of the evaluated patients had been diagnosed with multifocal desmoplastic fibromas. The lower limb was the most involved body area, accounting for a total of 87 desmoplastic fibromas (47%) [1,3,5,7,12,13,16,17,18,19,20,21,22,23,30,32,33,34,35,37,40,44,45,47,50,52,54,56,58,59,62,63,66,69,70,72,74,76,77,78,79,80,100,101,102,108]. The femur was the most affected single bone, as it alone was the location of 33 lesions (18%) [1,3,5,12,13,16,17,19,21,23,30,32,33,34,35,37,45,52,57,63,66,70,74,77,108]. Forty-eight lesions (26%) were located in the upper limb [1,3,5,11,13,15,17,20,30,32,33,34,35,36,39,42,46,48,51,53,55,64,65,67,73,81,89,92,93,94,96,104,105]. The mandible was the second most involved bone, with 27 lesions (14%) [14,30,33,35,40,43,68,83,84,86,87,88,90,96,97,100,103]. A total of 17 cases (9%) were documented with desmoplastic fibromas in their spine [31,38,49,60,75]. Finally, six lesions (3%) were localized in patients’ skull bones, including the zygoma [30,91,98,106], while the ribs were the site of two more neoplasms (1%) [82,102]. The distribution of desmoplastic fibromas in our study is graphically summarized in Figure 5. 

In two cases, described in separate case reports, desmoplastic fibromas (lesions localized to the femur and pelvis) developed in patients with fibrous dysplasia.

### 3.4. Clinical Picture: Pain, Swelling, Pathological Fractures

Data on the clinical presentation of bone desmoplastic fibromas were reported in 92 articles, covering a total of 161 patients [1,3,5,7,11,12,13,16,18,20,21,22,23,32,33,34,35,36,37,38,39,40,41,42,43,44,45,47,48,49,50,51,52,53,54,55,56,57,58,59,60,61,62,63,64,65,66,67,68,69,70,71,72,73,74,75,76,77,78,79,80,81,82,83,84,85,86,87,88,89,90,91,92,93,94,95,96,98,99,100,101,102,103,104,105,106]. One hundred seventeen patients (73%) experienced local pain, whereas 44 (27%) did not. In these latter cases, the diagnostic pathway was initiated by localized swelling, pathologic fractures, or lesions diagnosed incidentally during investigations of trauma or other diseases. No difference in pain rates could be found between lesions localized in the upper and lower limbs. In the mandible, only 23% of the lesions were painful [14,30,33,35,39,43,68,83,84,86,87,88,90,96,97,99,103]. Therefore, pain was significantly less common in mandibular desmoplastic fibromas compared to lesions arising from other anatomical districts (Fisher’s exact test; *p* < 0.0001). Swelling was detected in 86 patients (53%) [1,3,5,7,11,12,13,14,16,18,20,21,22,23,32,33,34,35,36,37,38,39,40,41,42,43,44,45,47,48,49,50,51,52,53,54,55,56,57,58,59,60,61,62,63,64,65,66,67,68,69,70,71,72,73,74,75,76,77,78,79,80,81,82,83,84,85,86,87,88,89,90,91,92,93,94,95,96,98,99,100,101,102,103,104,105,106]. Pathological fractures occurred in 17 cases (11%) [11,16,30,32,33,35,42,53,58,61,92]. Most of them occurred in the long bones of the upper or lower limb.

### 3.5. Lesions’ Size

The size of lesions was reported in 44 articles, providing information on 113 cases. The mean larger diameter of all lesions was 7.3 cm (0.8–19.0) [1,19,20,22,23,34,38,40,41,42,43,47,48,51,62,63,64,65,67,68,69,70,71,72,76,77,81,82,83,84,85,86,89,92,93,94,98,99,103,104,105,106,108].

### 3.6. Surgical Treatment

The surgical treatment of choice was intralesional curettage in 70 cases [1,3,5,7,12,13,16,17,19,20,30,31,32,33,34,35,36,37,38,39,40,41,42,45,51,55,57,64,66,68,69,72,74,80,86,87,92,96,100,108]. Wider resections were performed in 112 cases [1,3,5,11,14,15,18,21,22,23,30,31,32,33,34,35,36,37,38,39,40,41,43,44,46,47,48,49,50,52,53,54,56,58,59,60,61,62,63,65,67,70,71,73,75,76,77,78,79,81,82,83,84,85,88,89,90,91,93,94,95,97,98,99,101,102,103,104,105,106,107]. Amputations were carried out in 4 patients, all with large acral lesions of the upper or lower limb [3,34,39,54]. Finally, one case within a case series did not receive surgical treatment, but was included in our dataset as it could not be identified within the series [30]. A schematic resume of all the surgical approaches and eventual reconstructions included in our study is displayed in Table 3.

### 3.7. Post-Operative Follow-Up and Local Recurrence

**Figure 6:**
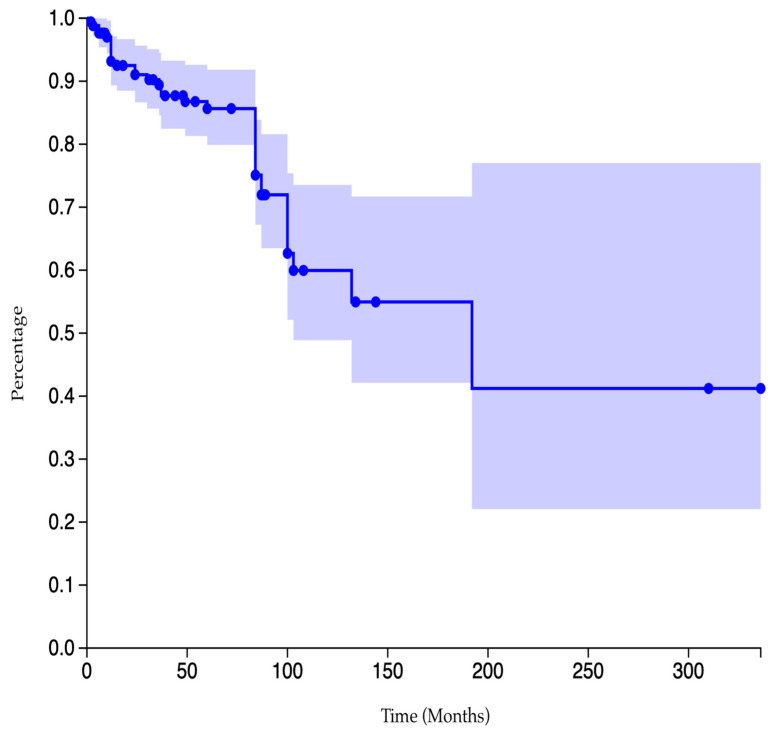
A Kaplan–Meier survival graph showing the global cumulative recurrence-free rate of patients after the diagnosis of bone desmoplastic fibromas and surgical treatment.

The post-operative follow-up was exhaustively described in 171 of 187 cases. Their mean post-operative follow-up was 65.3 months (6–336) [1,3,5,7,11,13,14,15,16,17,18,21,22,23,30,31,32,33,34,35,36,37,38,39,40,41,42,43,44,45,46,47,48,49,50,51,52,53,54,55,58,59,61,62,63,64,65,66,67,68,69,70,72,73,74,75,76,78,79,80,81,84,86,87,88,89,90,91,92,93,94,95,97,98,99,100,101,102,103,104,105,106,108]. Among them, 37 patients experienced local recurrences, resulting in an overall estimated recurrence rate of 21.6% [3,13,14,15,17,21,30,31,32,33,38,42,48,59,64,65,74,75,84,87,88,100]. The Kaplan–Meier curve of cumulative data in our review is pictured in Figure 6.

**Figure 7:**
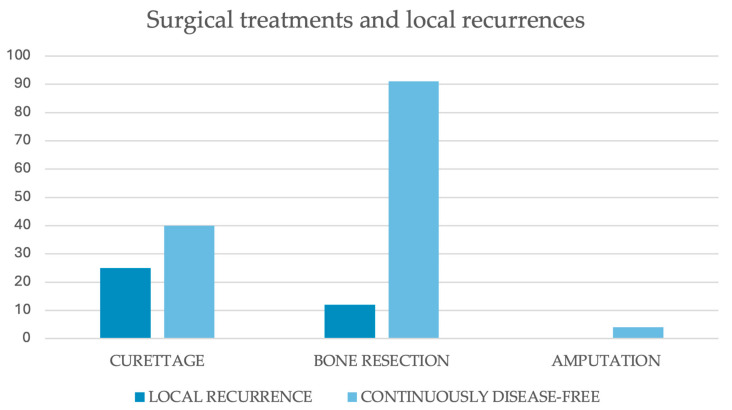
A graphic distribution of patients, sorted per surgical treatment and whether they had or not a local recurrence during their post-operative intercourse. Data were collected from the 172 patients who had follow-up details available. From left to right, clusters include patients treated with (left) curettage, (middle) bone resection, or (right) amputation. The dark blue column represents local recurrences, whereas the light blue column indicates patients who were continuously disease-free.

The recurrence rate among those treated with intralesional curettage was 38.5%. The rate of those who had bone resections was 11.6%, whereas none of the four cases described as being treated with amputations developed local secondary lesions (Table 1, Figure 7). According to a Chi-square test, cases treated with had a significantly higher local recurrence rate compared to those who received bone resection (*p* < 0.0001). 

In two cases (located in the femur and tibia), the local recurrence was associated with neoplastic degeneration and the diagnosis of secondary osteosarcoma [74,77].

## 4. Discussion

Since its discovery as a distinct pathology in the late 1950s, desmoplastic fibromas have been considered among the rarest benign tumors arising from bone tissue [1,2,3]. The number of desmoplastic fibromas reported in the literature has been estimated at hundreds. Our review confirms this perception, as only 187 cases of desmoplastic fibroma met our inclusion criteria [1,3,5,7,11,12,13,14,15,16,17,18,20,21,22,23,30,31,32,33,34,35,36,37,38,39,40,41,42,43,44,45,46,47,48,49,50,51,52,53,54,55,56,57,58,59,60,61,62,63,64,65,66,67,68,69,70,71,72,73,74,75,76,77,78,79,80,81,82,83,84,85,86,87,88,89,90,91,92,93,94,95,96,97,98,99,100,101,102,103,104,105,106,107,108]. Over 67 years, only 18 case series with information on their surgical treatment and postoperative follow-up have been identified [1,3,5,13,17,30,31,32,33,34,35,36,37,38,39,40,41]. The most numerous, with 27 cases, was published by Inwards et al. back in 1991 [30]. Only two other series included more than 10 cases [3,29], and 11 of them reported on only two or three patients [1,5,13,17,32,33,34,35,36,37,38,39,40,41]. The remaining evidence in modern literature comes from case reports. A total of 79 cases, accounting for 42.5% of the cases in our study, came from case reports [7,11,12,14,15,16,18,20,21,22,23,42,43,44,45,46,47,48,49,50,51,52,53,54,55,56,57,58,59,60,61,62,63,64,65,66,67,68,69,70,71,72,73,74,75,76,77,78,79,80,81,82,83,84,85,86,87,88,89,90,91,92,93,94,95,96,97,98,99,100,101,102,103,104,105,106,107,108]. A summary of the fragmented literature was necessary for an in-depth analysis of the epidemiology, clinical presentation, surgical treatments, and oncological outcomes of desmoplastic fibromas after surgery. 

Although our review does not support speculations on desmoplastic fibromas’ incidence, it provides epidemiological data regarding the gender and the age of patients diagnosed with the disease. A slight predilection for the female gender was observed in our study (104 males and 85 females), but no statistically significant gender discrepancy was found (*p* = 0.5266). Patients’ mean age at diagnosis was 24.3 (2–79). The tumor seemed to privilege the second and the third decades of life over other age groups [1,3,5,7,11,12,13,14,15,16,17,18,20,21,22,23,30,31,32,33,34,35,36,37,38,39,40,41,42,43,44,45,46,47,48,49,50,51,52,53,54,55,56,57,58,59,60,61,62,63,64,65,66,67,68,69,70,71,72,73,74,75,76,77,78,79,80,81,82,83,84,85,86,87,88,89,90,91,92,93,94,95,96,97,98,99,100,101,102,103,104,105,106,107,108]. 

Our review also allows us to clarify the distribution of desmoplastic fibromas through the human skeleton. The lower limb was the most involved anatomical district, accounting for 47% of all cases (87) included in our review [1,3,5,7,11,13,16,17,18,19,20,21,22,23,30,32,33,34,35,37,39,44,45,47,50,52,54,56,58,59,62,63,66,69,70,72,74,76,77,78,79,80,100,101,102,108]. The femur, in particular, was the single bone to host the most lesions, with a total of 33 [1,3,5,13,15,17,19,21,23,30,32,33,34,35,37,45,52,58,63,67,70,74,77,108]. Cases of desmoplastic fibromas in the upper limb were more exiguous. Forty-eight lesions were diagnosed in this area, with almost half arising from the radius alone [1,3,5,11,13,15,17,20,30,32,33,34,35,36,39,42,46,48,51,53,55,64,65,67,73,81,89,92,93,94,96,104,105]. Apart from the appendicular skeleton, desmoplastic fibromas were also described in the central skeleton, with most cases located in the spine (17 cases, 9.0%) [31,38,49,60,75] and the mandible [14,30,33,35,40,43,68,83,84,86,87,88,90,96,97,99,103]. This latter, in particular, hosted up to 27 cases (14.4%), second only to the femur as the most common localization for desmoplastic fibromas. Overall, our findings suggest that desmoplastic fibromas should be considered primarily in the differential diagnosis of primary osteolytic bone lesions in young adults, especially if arising from certain bones. The risk should be regarded as higher for femurs, pelvis, feet, tibias, and radius in the limbs, and the mandible and the spine in the central skeleton. Contrarily, the onset of such tumors in the other bones, although possible and described in the literature, is less likely to occur [28,82,91,98,102,108]. 

From a clinical perspective, various combinations of signs and symptoms were observed in the case series and case report under investigation. In total, 73% of the examined patients reported local soreness, whereas the remaining 27% did not complain of any pain. Most desmoplastic fibromas are thereby painful, especially those arising from limbs and spine [1,3,5,7,11,12,13,14,16,18,20,21,22,23,32,33,34,35,36,37,38,39,40,41,42,43,44,45,46,47,48,49,50,51,52,53,54,55,56,57,58,59,60,61,62,63,64,65,66,67,68,69,70,71,72,73,74,75,76,77,78,79,80,81,82,83,84,85,86,87,88,89,90,91,92,93,94,95,96,98,99,100,101,102,103,104,105,106]. Exception is given to mandibular lesions, which generally present as indolent nodules or alterations of the anatomical profile of the involved bone [14,30,33,35,40,43,68,83,84,86,87,88,90,96,97,99,103]. Swelling or local deformities were also common findings, visible in more than half of the investigated cases (53%) [1,3,5,7,11,12,13,14,16,18,20,21,22,23,32,33,34,35,36,37,38,39,40,41,42,43,44,45,47,48,49,50,51,52,53,54,55,56,57,58,59,60,61,62,63,64,65,66,67,68,69,70,71,72,73,74,75,76,77,78,79,80,81,82,83,84,85,86,87,88,89,90,91,92,93,94,95,96,98,99,100,101,102,103,104,105,106]. Despite the high incidence of pain and swelling, which might be wake-up calls to start the diagnostic pathway for most desmoplastic fibromas before massive degeneration of the host bone, up to 11% of investigated patients developed pathological fractures before surgical treatment. All fractures occurred in neoplasms localized in the lower or upper limbs, indicating a higher risk in the acral areas compared to the central parts of the body [11,16,30,32,33,35,42,53,58,61,92]. Even though pathological fractures could theoretically spread tumor cells, they were not associated with poorer oncological outcomes nor increased recurrence rates after surgery [110]. Nonetheless, physicians should pursue an early diagnosis to limit the increase in size of lesions and the onset of pathological fractures, as both eventualities could complicate surgical treatments [109]. 

Surgery represents the treatment of choice for desmoplastic fibromas and has been proven to eradicate the disease in the majority of treated cases [1,3,5,7,11,13,14,15,16,17,18,21,22,23,30,31,32,33,34,35,36,37,38,39,40,41,42,43,44,45,46,47,48,49,50,51,52,53,54,55,58,59,61,62,63,64,65,66,67,68,69,70,72,73,74,75,76,78,79,80,81,84,86,87,88,89,90,91,92,93,94,95,97,98,99,100,102,104,105,106,108]. Limb-sparing surgery represents the first-line treatment for most benign and locally aggressive bone tumors, including desmoplastic fibromas. On the contrary, amputations should be considered a last resort, reserved for acral lesions and complex cases. Both curettage [1,3,5,7,12,13,16,17,19,20,30,31,32,33,34,35,36,37,38,39,40,41,42,45,51,55,57,64,66,68,69,72,74,80,86,87,92,96,100,108] and focal bone resections [1,3,5,11,14,15,18,21,22,23,30,31,32,33,34,35,36,37,38,39,40,41,43,44,46,47,48,49,50,52,53,54,56,58,59,60,61,62,63,65,67,70,71,73,75,76,77,78,79,81,82,83,84,85,88,89,90,91,93,94,95,97,98,99,101,102,103,104,105,106,107] have been described and reported in large numbers in our review. Curettage can be considered for small-sized masses or lesions that preserve the continuity of the surrounding cortical bones. The relatively low invasiveness of such an approach can also be strategic in complex anatomical settings. In the case of meta-epiphyseal involvement, curettage alone or followed by grafting or cement filling can restore bone continuity and resistance, while preserving the native articulation. In young skeletally immature patients, who represent a significant proportion of those included in our review, curettage also allows for sparing of growth plates, thereby avoiding or limiting the risk of postoperative deformities [108,109,110,111,112]. Despite these advantages, intralesional curettage was burdened by a considerable risk of post-operative recurrence, as high as 38.5% in our review [3,13,14,15,17,21,30,31,32,33,38,42,48,59,64,65,74,75,84,87,88,100]. This risk could be theoretically reduced by implementing adjuvant strategies, such as high-speed burr, phenol, or intraoperative cryotherapy, to eliminate microscopic tumor residuals and free up surgical beds [113,114]. Larger bone resections, aimed at complete removal of the disease with wide margins, have been associated with significantly lower recurrence rates (11.6%) [1,3,5,11,14,15,18,21,22,23,30,31,32,33,34,35,36,37,38,39,40,41,43,44,46,47,48,49,50,52,53,54,56,58,59,60,61,62,63,65,66,70,71,73,75,76,77,78,79,81,82,83,84,85,88,89,90,91,93,94,95,97,98,99,101,102,103,104,105,106,107]. Our findings confirm the local aggressiveness and the tendency of desmoplastic fibromas to recur if not properly eradicated. Wide bone resections should be considered the treatment of choice when feasible, taking into account tumor location, patient age, and the overall clinical picture. Curettage, for its part, should be considered an alternative for small lesions, those located in the metaepiphyseal region with preserved cortical bone, and in skeletally immature patients.

We acknowledge that our study has some limitations. The rarity of desmoplastic fibromas limited the number of case series and the size of their cohorts, thereby limiting the reliability and significance of available data. Moreover, a large share of the examined data came from case reports, reducing the level of evidence in our casuistry. 

Beyond these limitations, our research provides an unprecedented overview of the demographics, localization, and clinical presentation of desmoplastic fibromas. Furthermore, our review evaluated the most commonly used surgical treatments for these neoplasms, assessing their effectiveness in terms of recurrence-free survival during postoperative follow-up. 

This review summarizes the modern literature, aiming to guide orthopedic oncologists in their approach to desmoplastic fibromas of the bone, from the clinical approach to identify the neoplasm to the surgical approach of choice to eradicate the disease.

## 5. Conclusions

To this date, the literature lacks large-scale studies on the clinical presentation and the surgical treatment of desmoplastic fibromas of the bone. Their introduction, as well as multicentric studies, would be advisable to overcome the low incidence of desmoplastic fibromas. However, tens of case reports and small case series have already been described in the literature, and our review first summarizes their outcomes. Physicians should consider desmoplastic fibromas in the differential diagnosis of locally aggressive bone lesions, particularly if located in relatively high-incidence sites such as the femur, pelvis, or mandible. Orthopedic surgeons should carefully choose the limb-sparing treatment of choice between wide resections and intralesional curettage, being aware of the loss of bone stock associated with the former and the risk of local recurrence with the latter.

## Figures and Tables

**Table 1:** A schematic resume of the data recorded from the single case series and (in the last row) a summary of all the case reports included in our review.

Article	N	Mean Age (Y)	Tumor Location	Mean Size (mm)	Painful	Swelling	Surgery	Recurr. Rate	Mean FU (M)
Inwards et al. (1991) [30]	27	24	7Mandible, 5Radius, 3Humerus, 3Pelvis, 3Femur, 2Foot, 1Skull, 1Zygoma, 1Scapula, 1Tibia	-	18 (67%)	18 (67%)	10 Curettage, 16 Resection (1 Wait-and-see)	9 (35%)	84
Evans et al. (2014) [3]	13	12	3Radius, 2Pelvis, 2Tibia, 2Metacarpal, 2Talus, 1Calcaneum, 1Fibula	-	11 (85%)	6 (46%)	6 Curettage 6 Resection 1 Amputation	2 (15%)	87
Yin et al. (2014) [31]	12	37.5	12Spine (5C 3T 3L 1S)	-	-	-	12 Resection	1 (8%)	49
Nilsonne & Gothlin (1969) [32]	9	33.3	5Pelvis, 2Scapula, 1Tibia, 1Femur,	-	8 (89%)	0	9 Curettage	1 (11%)	103
Gebhardt et al. (1985) [33]	8	25	3 Tibia, 1Radius, 1Foot, 1Pelvis, 1Femur, 1Mandible	-	6 (75%)	2 (25%)	6 Curettage 2 Resection	4 (50%)	100
Crim et al. (1989) [17]	7	22.7	5Femur, 1Pelvis, 1Radius	-	-	-	3 Curettage 4 Resection	3 (43%)	54
Bertoni et al. (1984) [1]	6	26.8	2Fibula, 1Scapula, 1Humrus, 1Femur, 1Calcaneus	88	4 (67%)	4 (67%)	2 Curettage 4 Resection	0	134
Ferri et al. (2013) [41]	3	2.3	3Mandible	46	0	3 (100%)	3 Resection	0	89
Rastogi et al. (2008) [34]	3	16.7	1Radius, 1Foot, 1Femur	105	3 (100%)	2 (67%)	1 Curettage 1 Resection 1 Amputation	0	88
Tanwar et al. (2017) [5]	3	36.7	2Femur, 1Humerus	-	3 (100%)	2 (67%)	3 Curettage	0	44
Lagace et al. (1979) [35]	3	19.3	1Femur, 1Radius, 1Mandible	-	3 (100%)	0	3 Resection	0	15
Dahlin et al. (1964) [36]	2	18.5	2Radius	-	2 (100%)	2 (100%)	2 Curettage	0	310
Lau et al. (2013) [38]	2	22.5	2Spine (C)	24	2 (100%)	0	2 Resection	2 (100%)	37
Young et al. (1988) [13]	2	14	1Radus, 1Femur	-	1 (50%)	1 (50%)	1 Curettage 1 Resection	1 (50%)	12
Hardy & Lehrer (1967) [39]	2	13.5	1Radius, 1Pelvis	-	1 (50%)	1 (50%)	1 Curettage 1 Amputation	0	39
Mahnken et al. (2001) [37]	2	44	1Femur, 1 Tibia	-	2 (100%)	0	1 Curettage 1 Resection	0	108
Bohm et al. (1996) [19]	2	30.5	2Femur, 1Tibia	111	2 (100%)	0	2 Resection	0	31.5
Woods et al. (2014) [40]	2	38.5	2Mandible	120	1 (50%)	1 (50%)	2 Resection	0	39
Case reports (1976–2025) [7,11,12,14,15,16,18,20,21,22,23,42,43,44,45,46,47,48,49,50,51,52,53,54,55,56,57,58,59,60,61,62,63,64,65,66,67,68,69,70,71,72,73,74,75,76,77,78,79,80,81,82,83,84,85,86,87,88,89,90,91,92,93,94,95,96,97,98,99,100,101,102,103,104,105,106,107,108]	79	26.5	15Femur, 13Mandible, 9Foot, 8Pelvis, 7Radius, 5Ulna, 4 Tibia, 4Humerus, 3Spine, 3Skull, 2Rib, 2Hand, 2Scapula, 1Clavicle, 1Zygoma	70	50 (66% *)	44 (57% *)	25 Curettage 54 Resection 1 Amputation	14 (22% *)	42.5 *

Recurr. = Recurrence; FU = Follow-Up; (Y) = Years; (M) = Months; (mm) = Millimeters; (C) = Cervical; (T) = Thoracic; (L) = Lumbar; (S) = Sacral; * = Data considering only the cases with details on clinical presentation or post-operative follow-up and local recurrence.

**Table 2:** The answers to all the queries of the JBI checklist for case series studies.

Authors	Q1	Q2	Q3	Q4	Q5	Q6	Q7	Q8	Q9	Q10
Inwards et al. (1991) [30]	Yes	Yes	Yes	Yes	Yes	Yes	Unclear	Unclear	Yes	NA
Evans et al. (2014) [3]	Yes	Yes	Yes	Yes	Yes	Yes	Yes	Yes	Yes	NA
Yin et al. (2014) [31]	Yes	Yes	Yes	Yes	Yes	Yes	Unclear	Unclear	Yes	NA
Nilsonne & Gothlin (1969) [32]	Yes	Yes	Yes	Yes	Yes	Yes	Yes	Yes	Yes	NA
Gebhardt et al. (1985) [33]	Yes	Yes	Yes	Yes	Yes	Yes	Yes	Unclear	Yes	NA
Crim et al. (1989) [17]	Yes	Yes	Yes	Yes	Yes	Yes	Unclear	Yes	Yes	NA
Bertoni et al. (1984) [1]	Yes	Yes	Yes	Yes	Yes	Yes	Yes	Yes	Yes	NA
Ferri et al. (2013) [41]	Yes	Yes	Yes	Yes	Yes	Yes	Yes	Yes	Yes	NA
Rastogi et al. (2008) [34]	Yes	Yes	Yes	Yes	Yes	Yes	Yes	Yes	Yes	NA
Tanwar et al. (2017) [5]	Yes	Yes	Yes	Yes	Yes	Yes	Yes	Yes	Yes	NA
Lagace et al. (1979) [35]	Yes	Yes	Yes	Yes	Yes	Yes	Yes	Yes	Yes	NA
Dahlin et al. (1964) [36]	Yes	Yes	Yes	Yes	Yes	Yes	Yes	Yes	Yes	NA
Lau et al. (2013) [38]	Yes	Unclear	Yes	Unclear	Yes	Yes	Yes	Yes	Yes	NA
Young et al. (1988) [13]	Yes	Unclear	Yes	Yes	Yes	Yes	Yes	Yes	Yes	NA
Hardy & Lehrer (1967) [39]	Yes	Yes	Yes	Unclear	Yes	Yes	Yes	Yes	Yes	NA
Mahnken et al. (2001) [37]	Yes	Unclear	Yes	Yes	Yes	Yes	Yes	Yes	Yes	NA
Bohm et al. (1996) [19]	Yes	Yes	Yes	Yes	Yes	Yes	Yes	Yes	Yes	NA
Woods et al. (2014) [40]	Yes	Unclear	Yes	Unclear	Yes	Yes	Yes	Yes	Yes	NA

NA = Not Applicable.

**Table 3:** Schematic summary of the surgical approaches described by the studies included in our review.

	Curettage	Bone Resections	Amputations
**Total number**	**70**	**112**	**4**
**Reconstruction**			
None/Not mentioned	43	55	-
Bone graft	25 23 Allograft 2 Autograft (ilium)	30 15 Allograft 15 Autograft (8 Fibula, 4 Rib, 3 Ilium)	-
Prosthesis	-	6 Prosthesis	-
Other	2 PMMA	1 Allograft-Prosthesis composite 20 Metallic stabilization	-
**Local adjuvants**	1 cryotherapy 1 heat ablation	1 cryotherapy	-
Local recurrence *	25/65 (38.5%)	12/103 (11.6%)	0/4

* Ratio of local recurrence among those who had information on their post-operative follow-up.

## Data Availability

The data that support the findings of this study are available from the corresponding author upon reasonable request.

## Supplementary Materials

The following supporting information can be downloaded at: https://www.mdpi.com/article/10.3390/cancers17213558/s1, PRISMA 2020 Checklist.

## Appendix A

**Search query strings:** [PubMed, Medline, EMBASE, Scopus] (Desmoplastic Fibroma) AND (Bone) AND (Surgery).

**Data researched on**: 1 August 2025.
